# Supplementary figures and images for: Orderly mitosis shapes interphase genome architecture
Source: eLife. 2026 Apr 21;14:RP108410. doi: 10.7554/eLife.108410 (PMC13099139; doi:10.7554/eLife.108410)

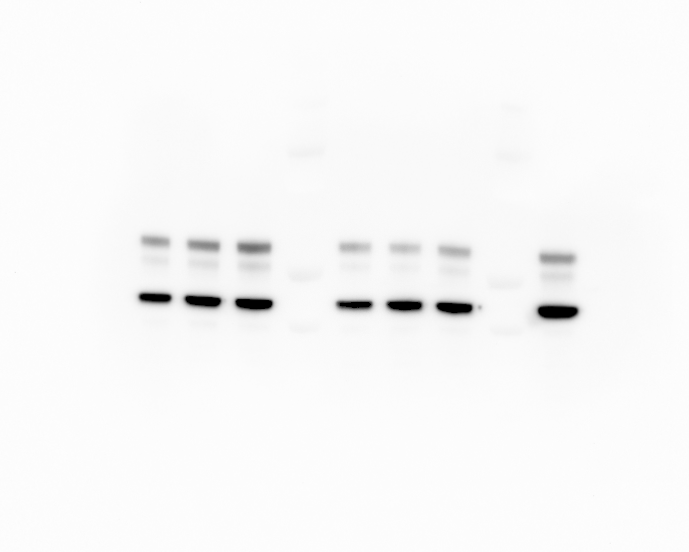

Supplement: Figure 3—figure supplement 1—source data 1. [file elife-108410-fig3-figsupp1-data1.zip › Figure 3-figure supplement 1b_source_data_beta_actin.raw16.tif]

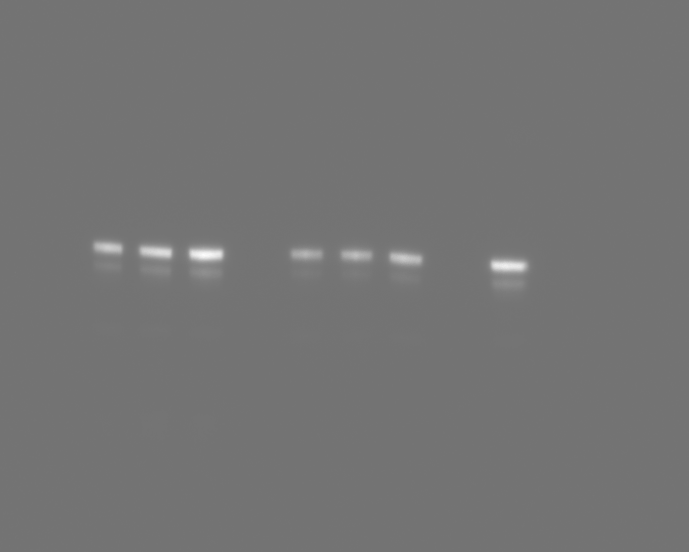

Supplement: Figure 3—figure supplement 1—source data 1. [file elife-108410-fig3-figsupp1-data1.zip › Figure 3-figure supplement 1b_source_data_NCAPH2.tif]

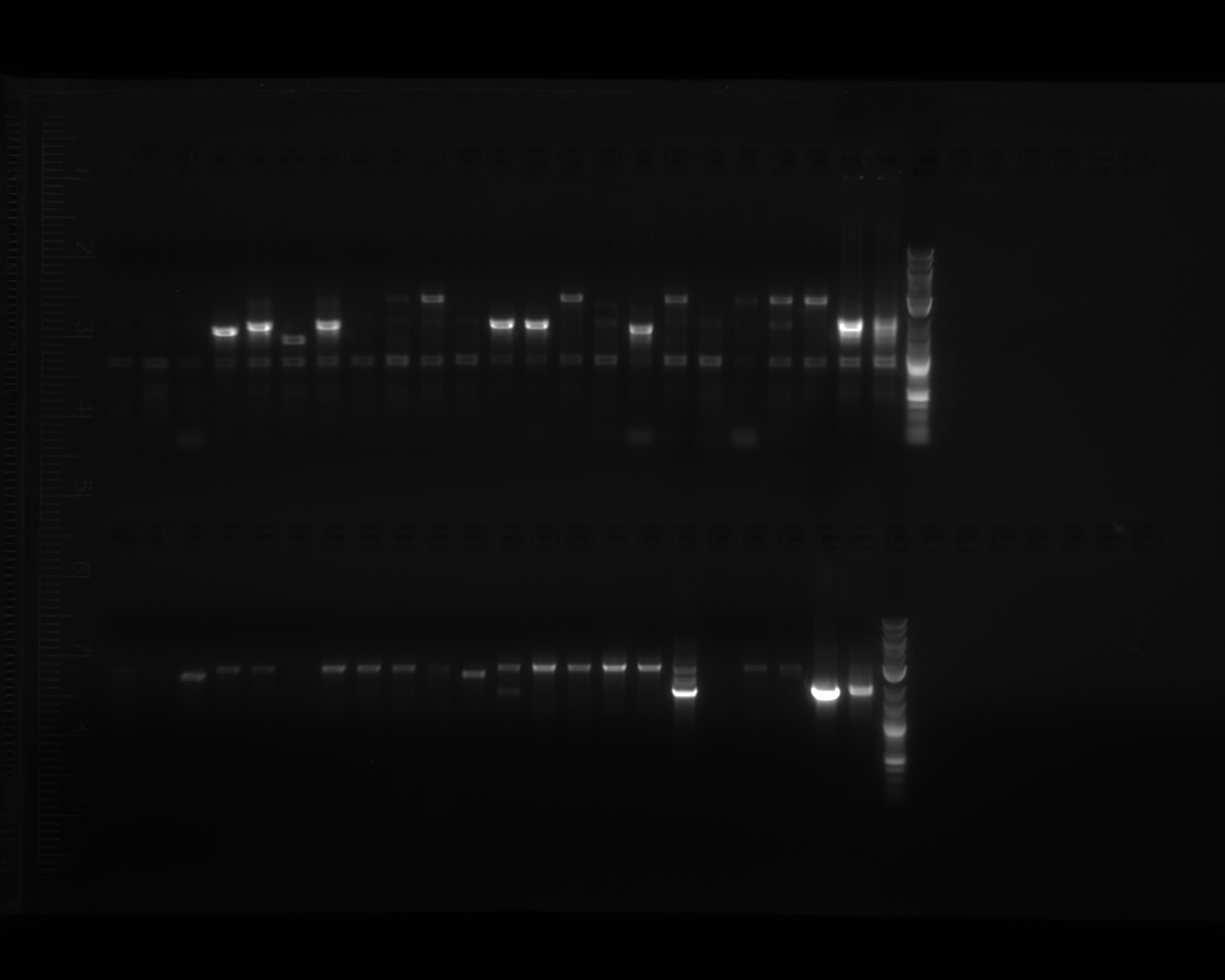

Supplement: Figure 4—figure supplement 1—source data 1. [file elife-108410-fig4-figsupp1-data1.zip › Figure_4_figure_supplement_1_source_data_1.tif]

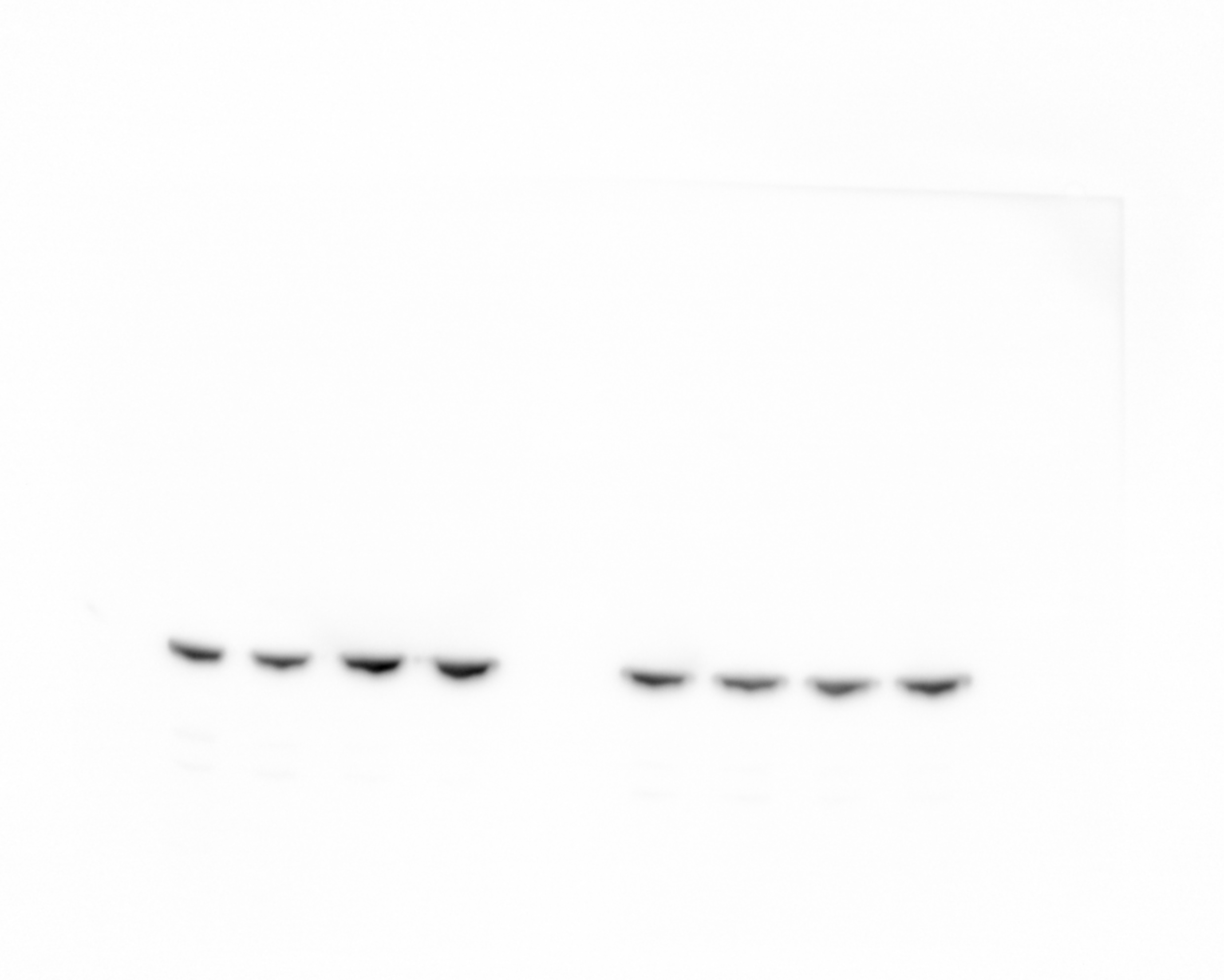

Supplement: Figure 4—figure supplement 2—source data 1. [file elife-108410-fig4-figsupp2-data1.zip › Figure_4_figure_supplement_2_c_and_d_source_data_anti_beta_actin_blot.tif]

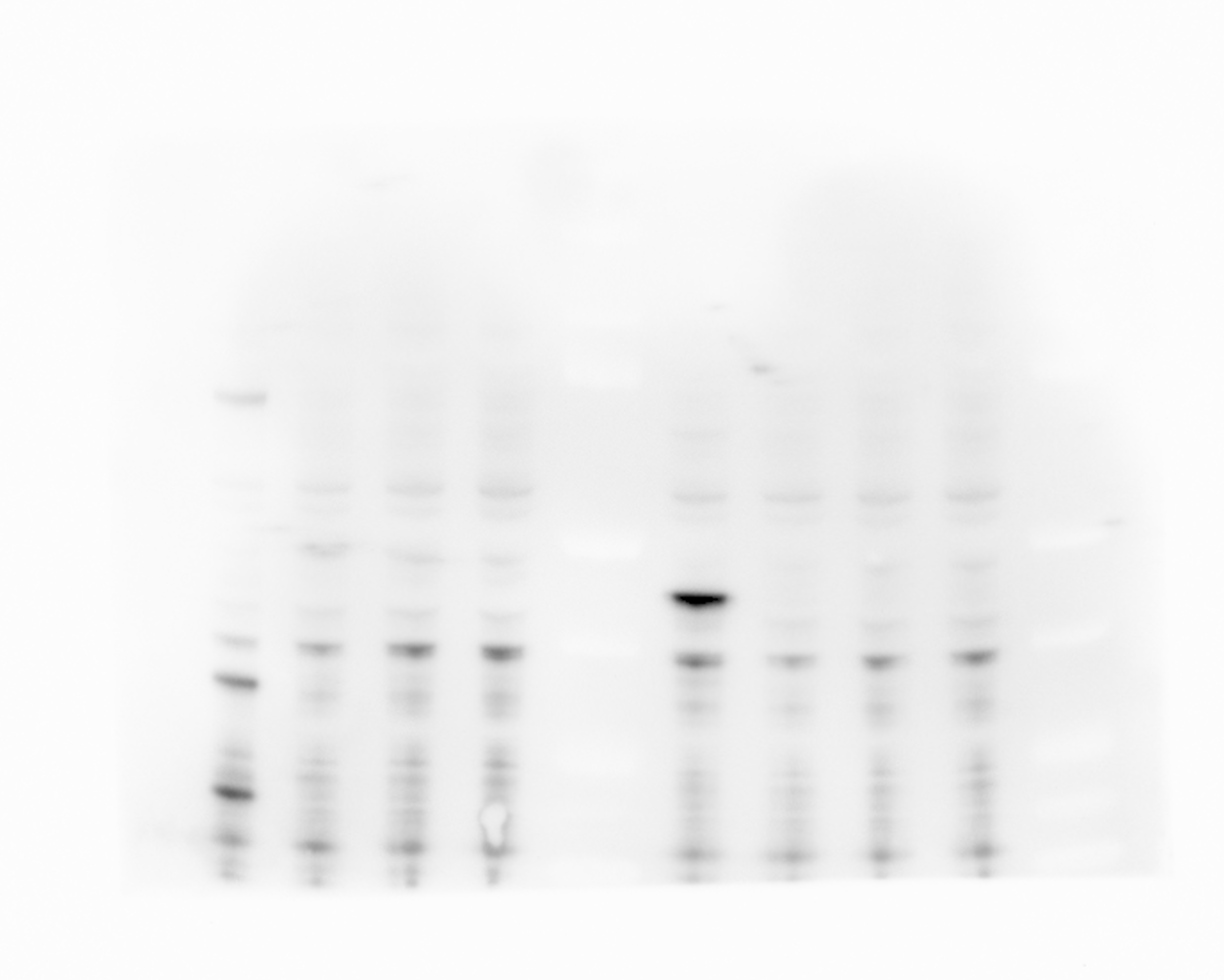

Supplement: Figure 4—figure supplement 2—source data 1. [file elife-108410-fig4-figsupp2-data1.zip › Figure_4_figure_supplement_2_c_and_d_source_data_anti_FLAG_blot.tif]

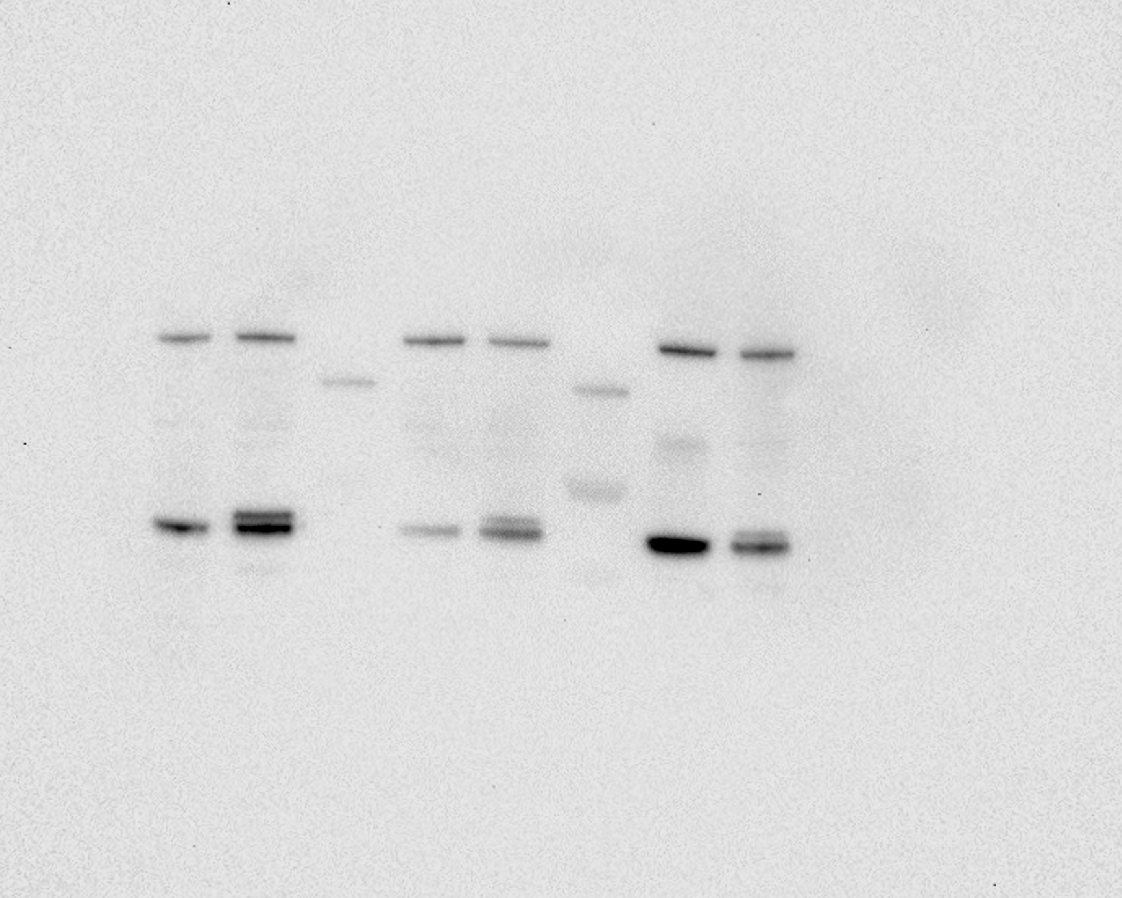

Supplement: Figure 4—figure supplement 2—source data 1. [file elife-108410-fig4-figsupp2-data1.zip › Figure_4_figure_supplement_2_e_source_data_anti_beta_actin_blot.tif]

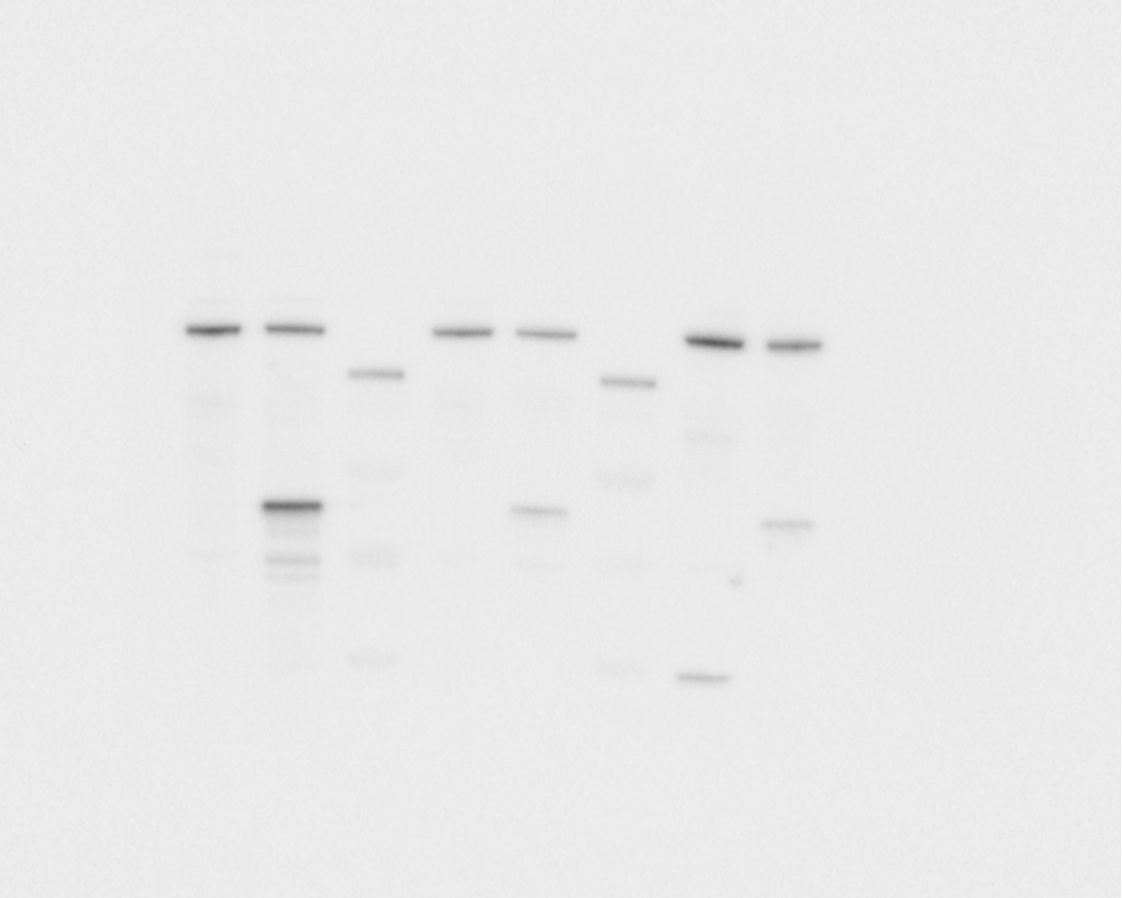

Supplement: Figure 4—figure supplement 2—source data 1. [file elife-108410-fig4-figsupp2-data1.zip › Figure_4_figure_supplement_2_e_source_data_anti_SPC24_blot.tif]

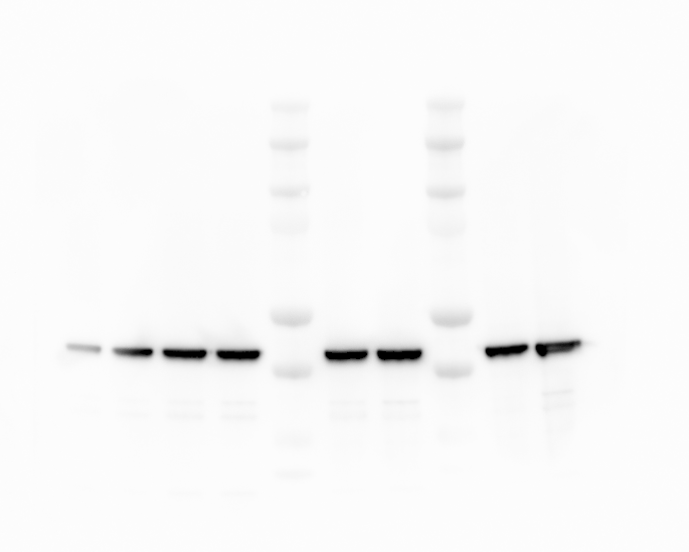

Supplement: Figure 4—figure supplement 2—source data 1. [file elife-108410-fig4-figsupp2-data1.zip › Figure_4_figure_supplement_2_f_source_data_anti_beta_actin_blot.tif]

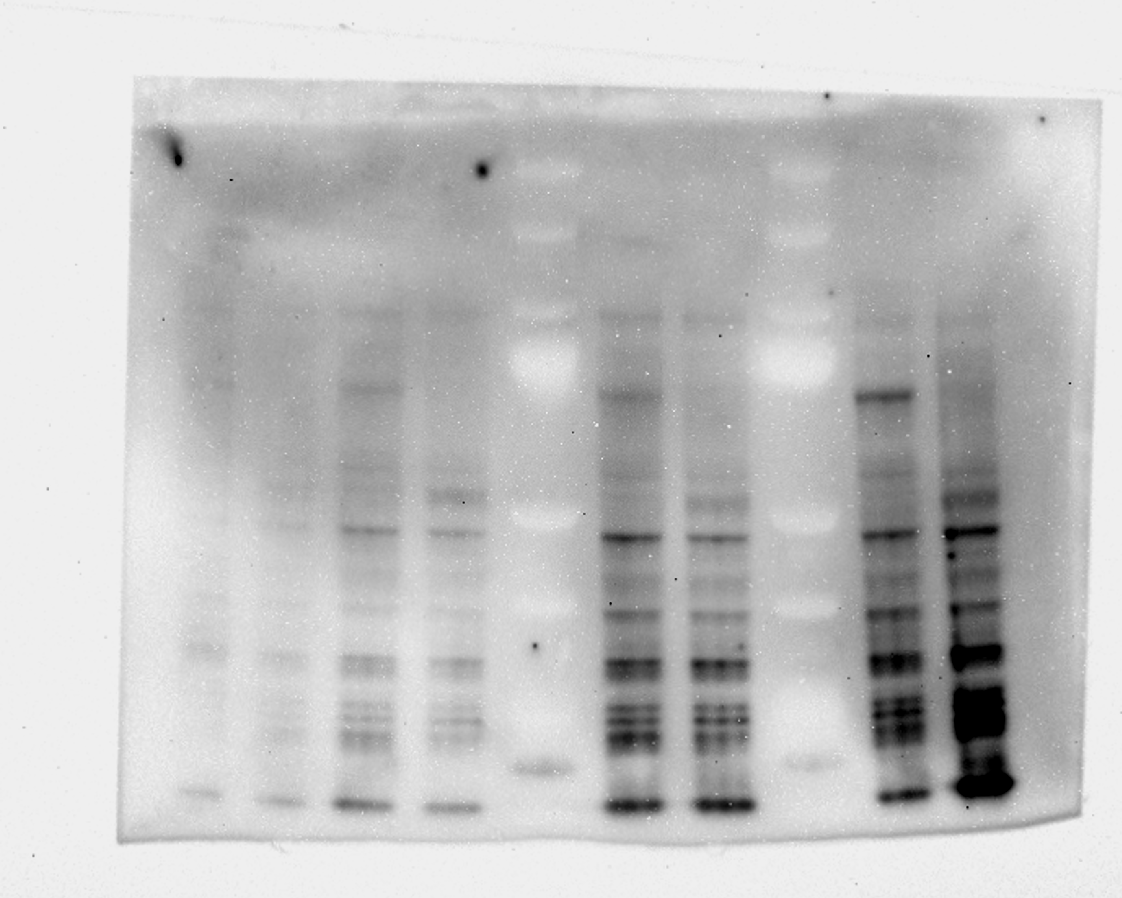

Supplement: Figure 4—figure supplement 2—source data 1. [file elife-108410-fig4-figsupp2-data1.zip › Figure_4_figure_supplement_2_f_source_data_anti_NUF2_blot.tif]

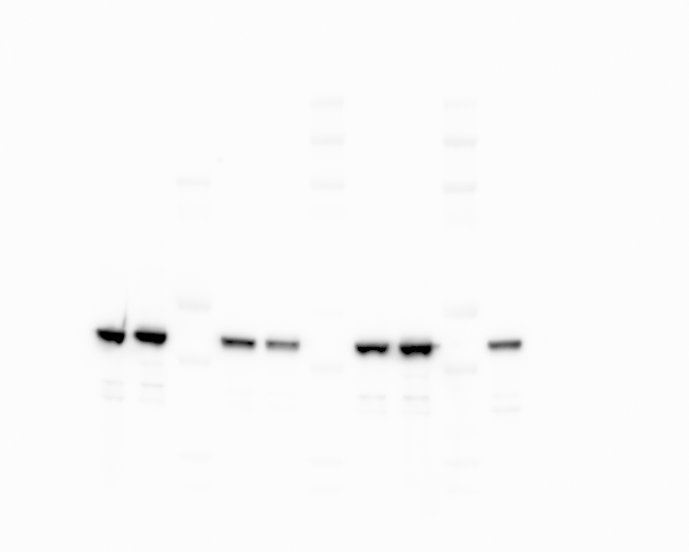

Supplement: Figure 4—figure supplement 3—source data 1. [file elife-108410-fig4-figsupp3-data1.zip › Figure_4_figure_supplement_3a_anti_beta_actin.tif]

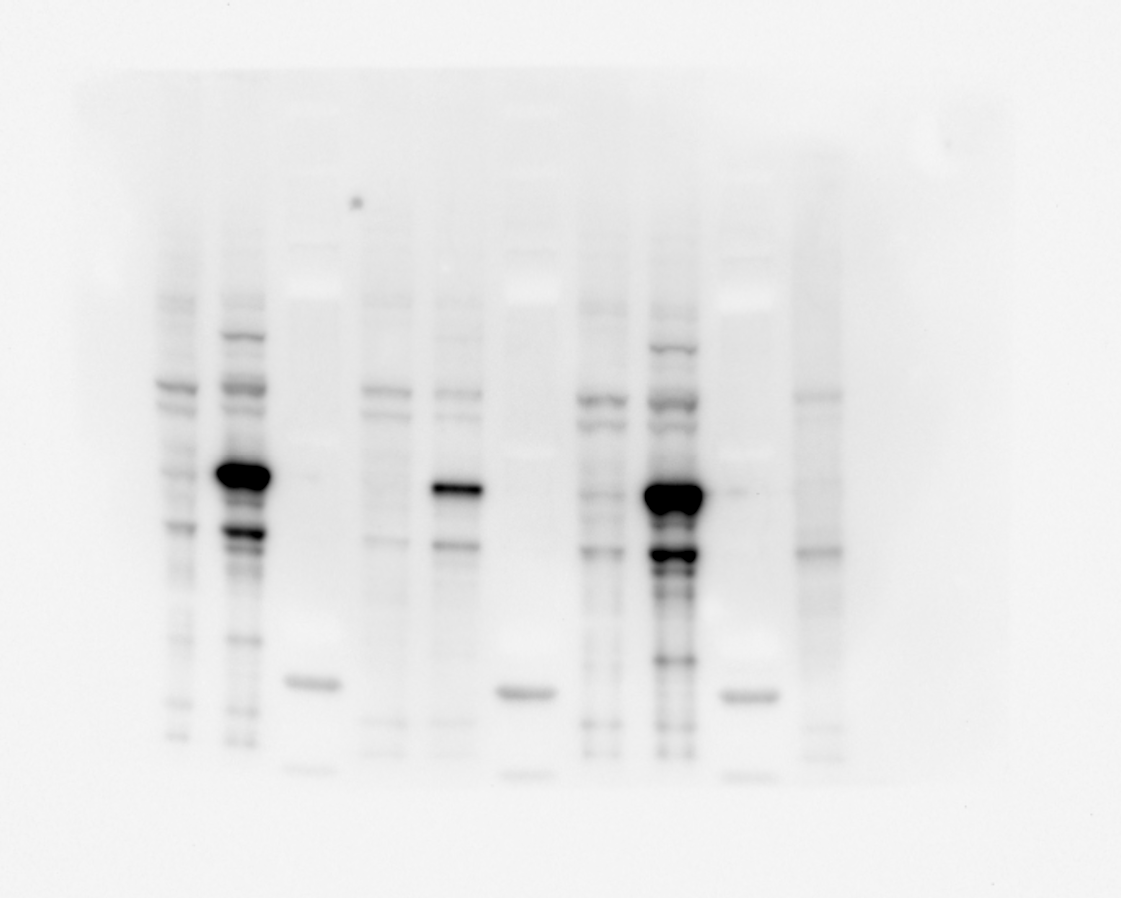

Supplement: Figure 4—figure supplement 3—source data 1. [file elife-108410-fig4-figsupp3-data1.zip › Figure_4_figure_supplement_3a_anti_FLAG.tif]

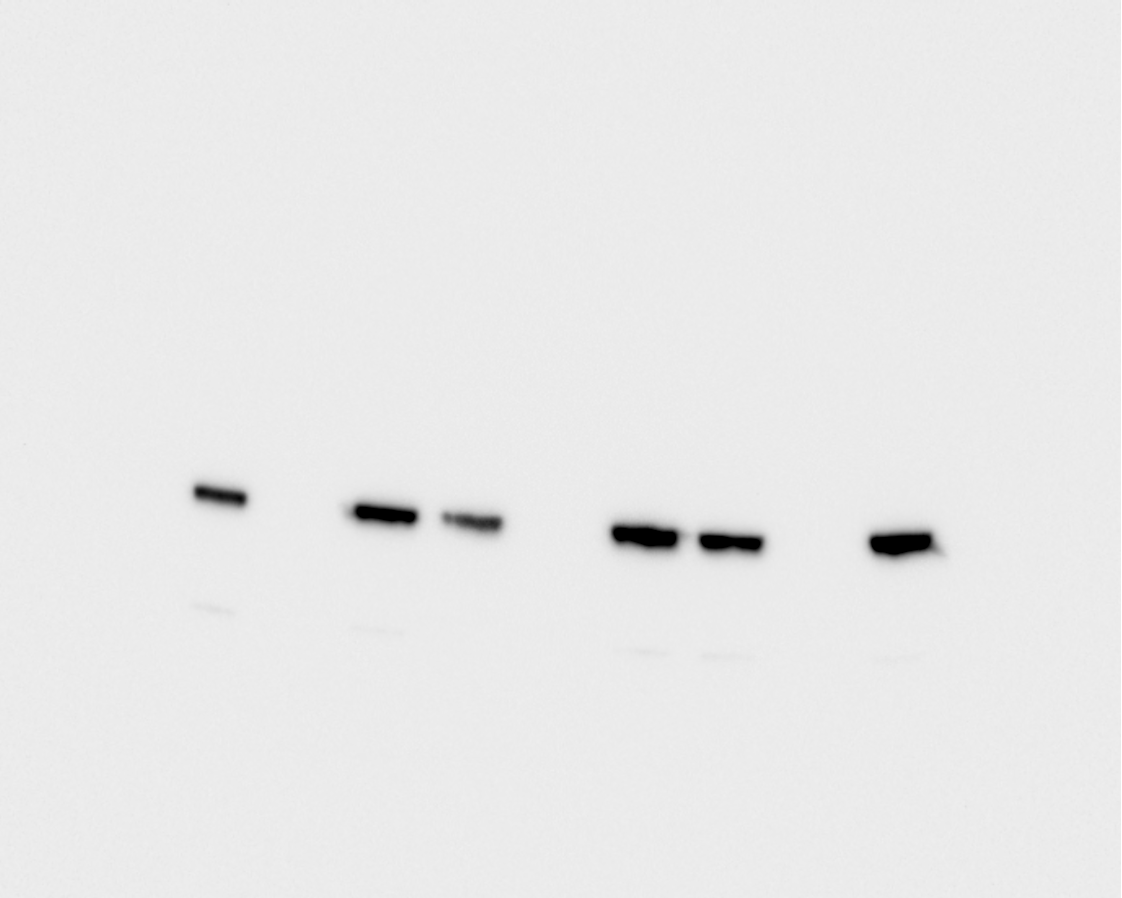

Supplement: Figure 4—figure supplement 3—source data 1. [file elife-108410-fig4-figsupp3-data1.zip › Figure_4_figure_supplement_3b_anti_beta_actin.tif]

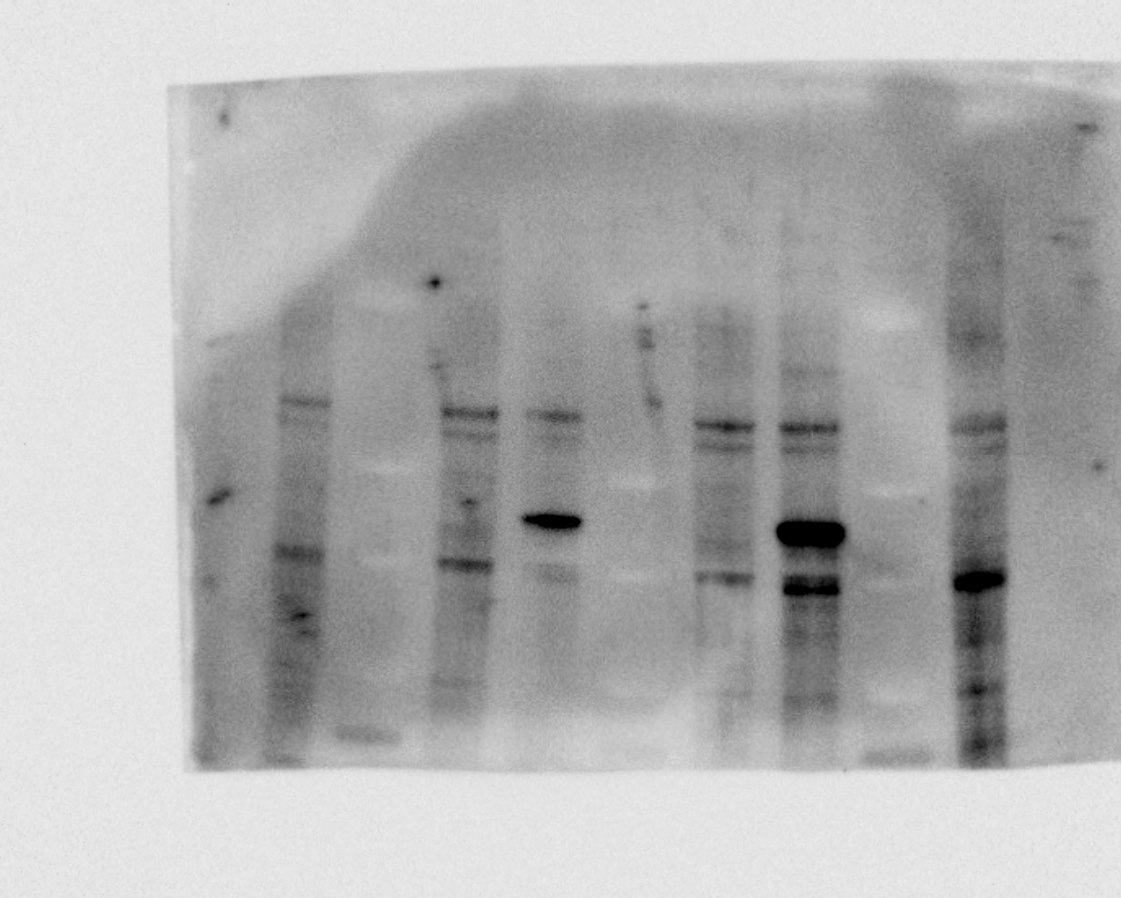

Supplement: Figure 4—figure supplement 3—source data 1. [file elife-108410-fig4-figsupp3-data1.zip › Figure_4_figure_supplement_3b_anti_FLAG.tif]

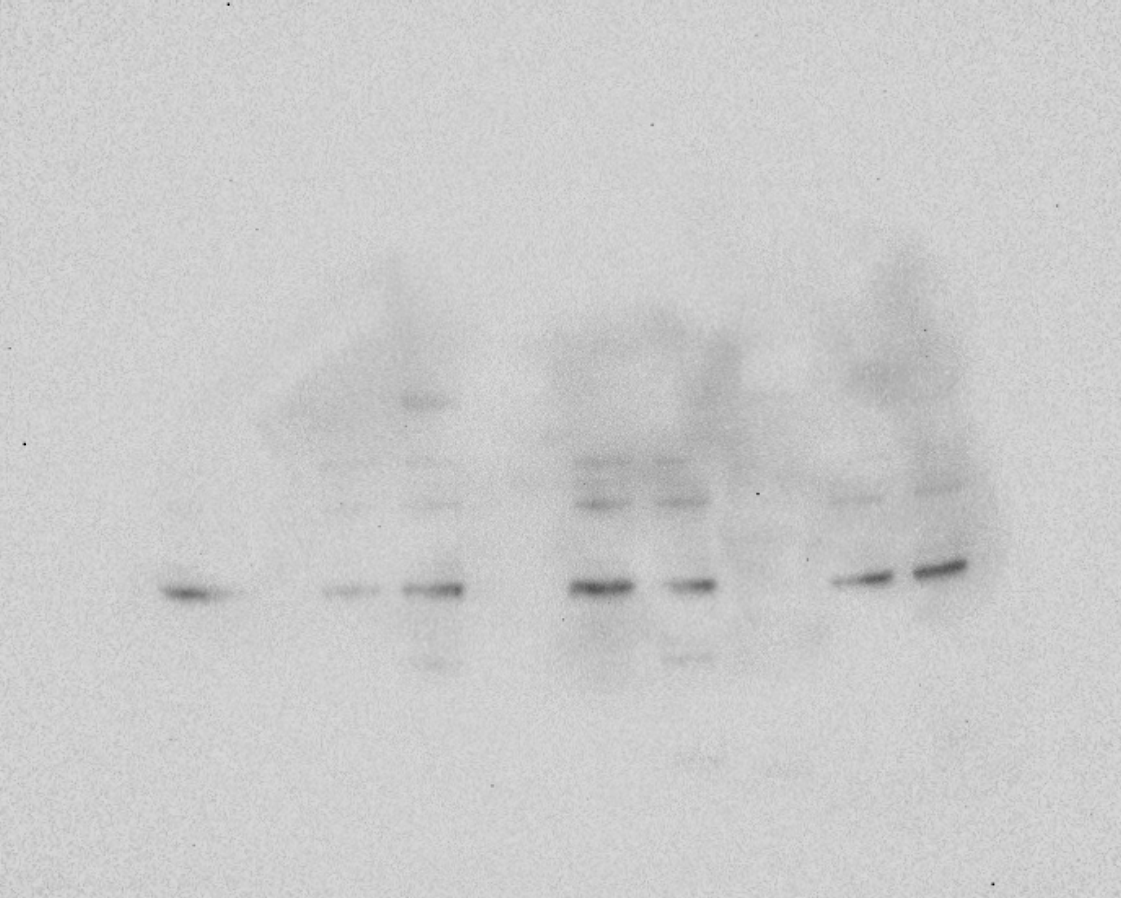

Supplement: Figure 4—figure supplement 3—source data 1. [file elife-108410-fig4-figsupp3-data1.zip › Figure_4_figure_supplement_3c_anti_beta_actin.tif]

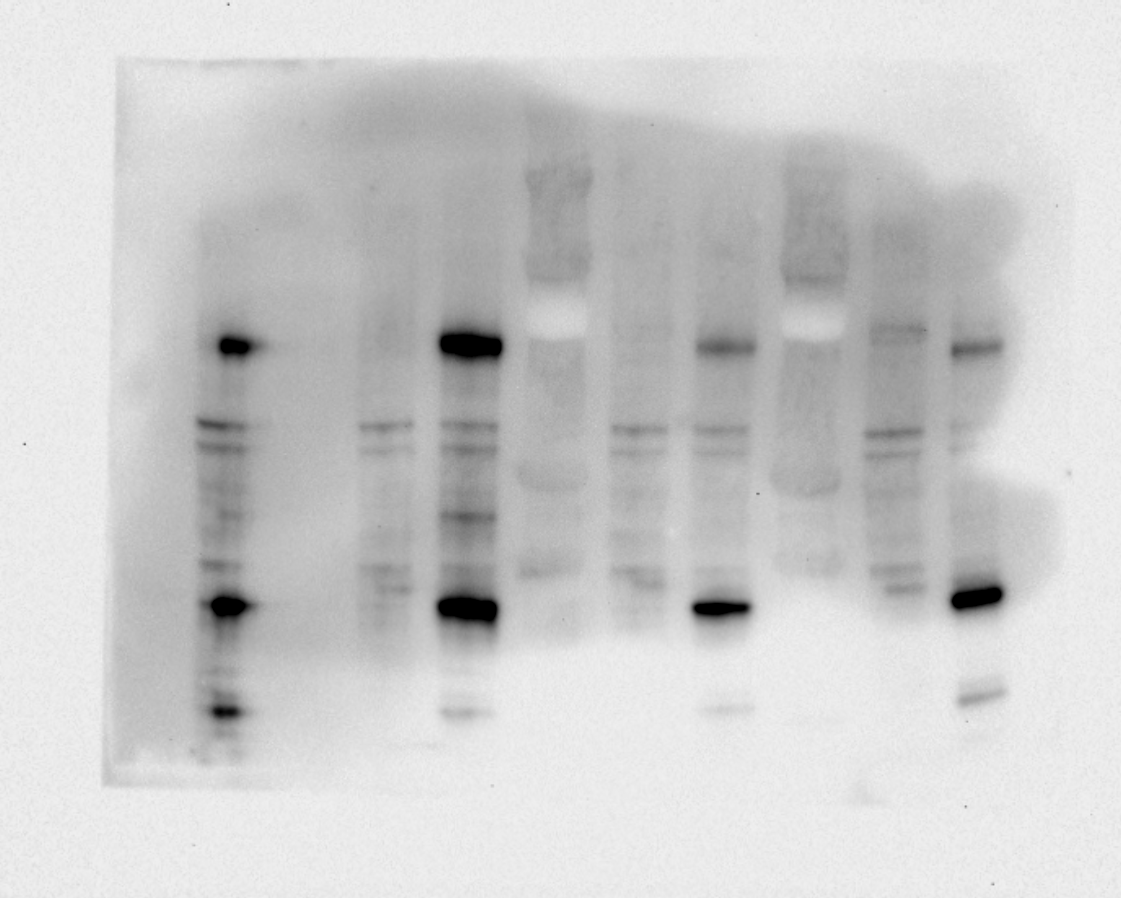

Supplement: Figure 4—figure supplement 3—source data 1. [file elife-108410-fig4-figsupp3-data1.zip › Figure_4_figure_supplement_3c_anti_FLAG.tif]

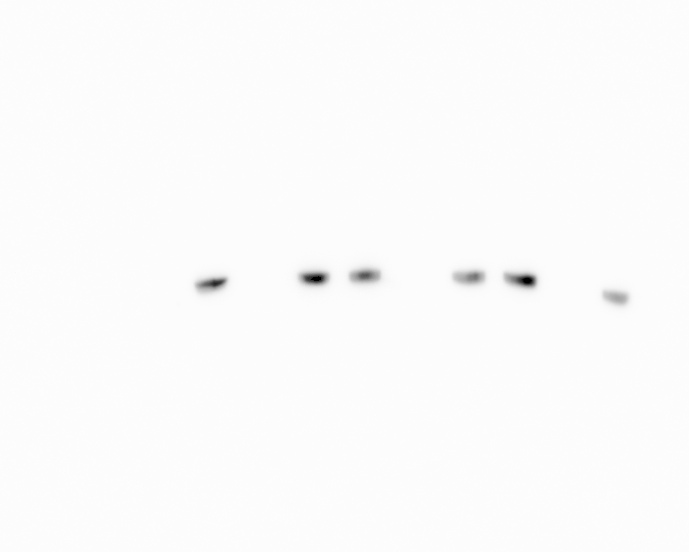

Supplement: Figure 4—figure supplement 3—source data 1. [file elife-108410-fig4-figsupp3-data1.zip › Figure_4_figure_supplement_3d_anti_beta_actin.tif]

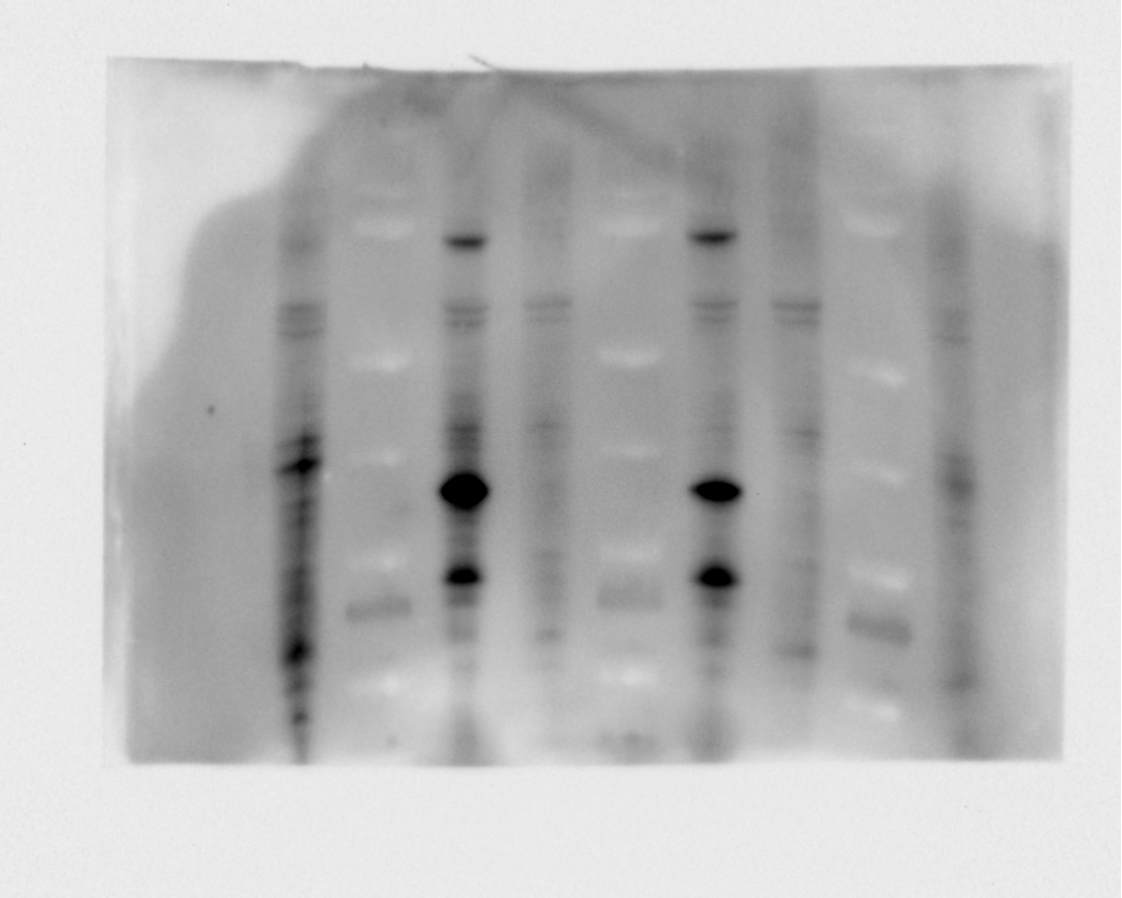

Supplement: Figure 4—figure supplement 3—source data 1. [file elife-108410-fig4-figsupp3-data1.zip › Figure_4_figure_supplement_3d_anti_FLAG.tif]
