# Supplementary material for: Orderly mitosis shapes interphase genome architecture
Source: eLife. 2026 Apr 21;14:RP108410. doi: 10.7554/eLife.108410 (PMC13099139; doi:10.7554/eLife.108410)
Supplement: Figure 3—figure supplement 1—source data 2. [file elife-108410-fig3-figsupp1-data2.zip › Figure_3_figure_supplement_1_source_data_2.pdf]

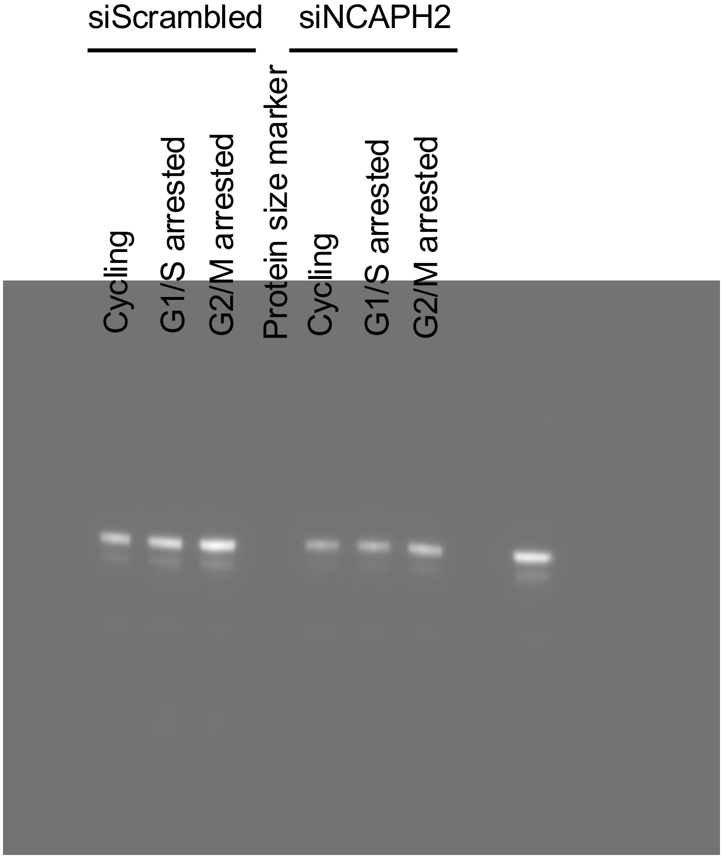

Blot incubated with anti NCAPH2 primary antibody

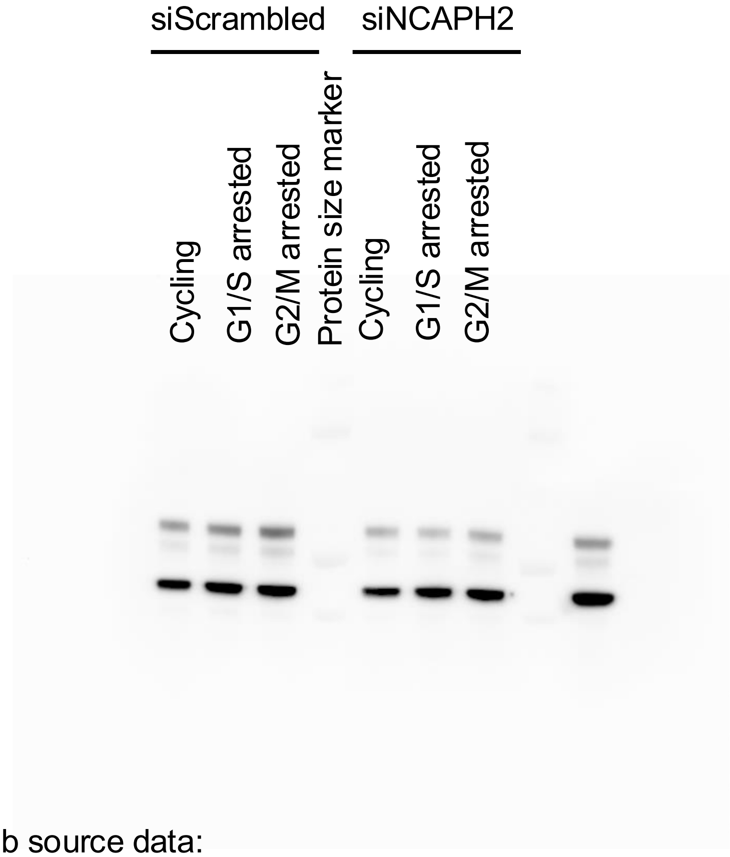

The same blot was incubated with anti β-actin primary antibody

Figure 3-figure supplement 1b source data:
Original western blot chemiluminescence images used for quantification of NCAPH2 protein level in cycling, G1/S or G2/M arrested cells upon siRNA knockdown of NCAPH2 as compared to siScrambled control. This membrane was incubated with NCAPH2 antibody (top) and β-actin antibody (bottom). Protein size markers loaded in the middle lane are largely invisible in the chemiluminescence images. Lanes that are not labelled in this blot are unrelated to S6b.
