# Supplementary material for: Orderly mitosis shapes interphase genome architecture
Source: eLife. 2026 Apr 21;14:RP108410. doi: 10.7554/eLife.108410 (PMC13099139; doi:10.7554/eLife.108410)
Supplement: Figure 4—figure supplement 1—source data 2. [file elife-108410-fig4-figsupp1-data2.zip › Figure_4_figure_supplement_1_source_data_2.pdf]

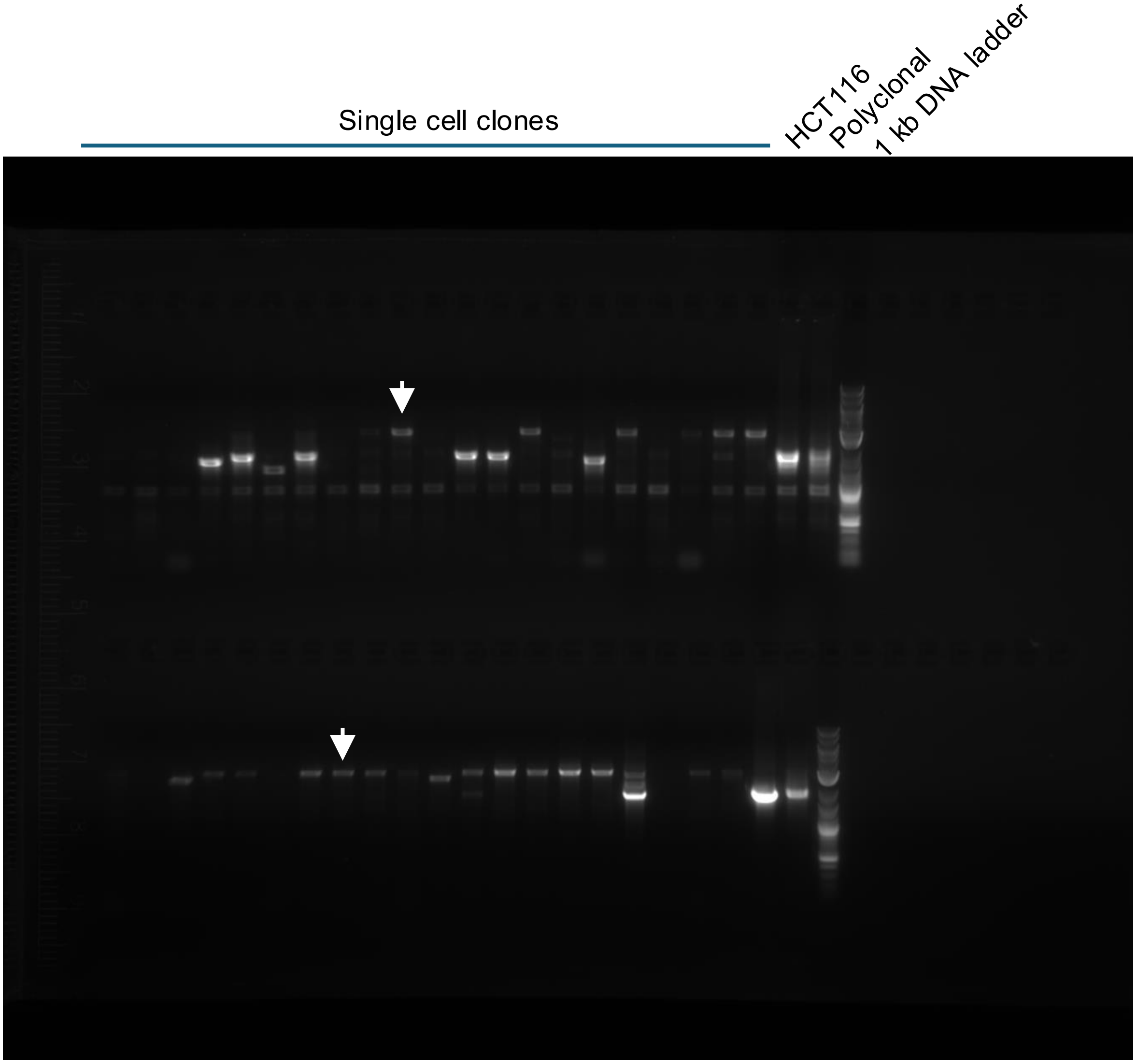

Figure 4-figure supplement 1c and d source data:
Original ethidium bromide-stained gel image showing PCR confirmation of dTAG tagging of both alleles of NUF2 (top) and SPC24 (bottom) based on the strategy explained in S7b and S7a respectively. White arrowheads indicate lane for specific clone N10 for NUF2 degron line and S8 for SPC24 degron line used for experiments. Genomic DNA used for PCR in each lane are indicated at the top of the image.
