# Supplementary material for: Orderly mitosis shapes interphase genome architecture
Source: eLife. 2026 Apr 21;14:RP108410. doi: 10.7554/eLife.108410 (PMC13099139; doi:10.7554/eLife.108410)
Supplement: Figure 4—figure supplement 2—source data 2. [file elife-108410-fig4-figsupp2-data2.zip › Figure_4_figure_supplement_2_source_data2.pdf]

Blot incubated with
anti FLAG
primary antibody

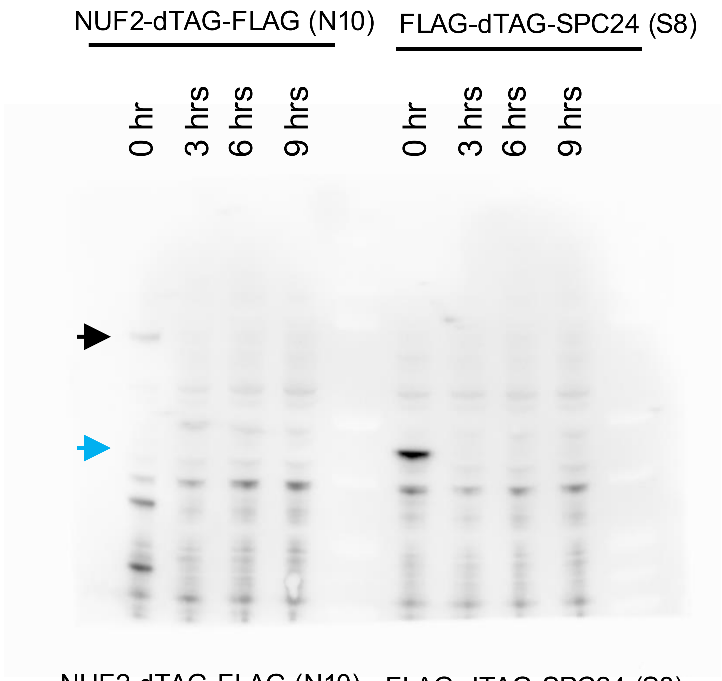

Blot incubated with
anti β-actin
primary antibody

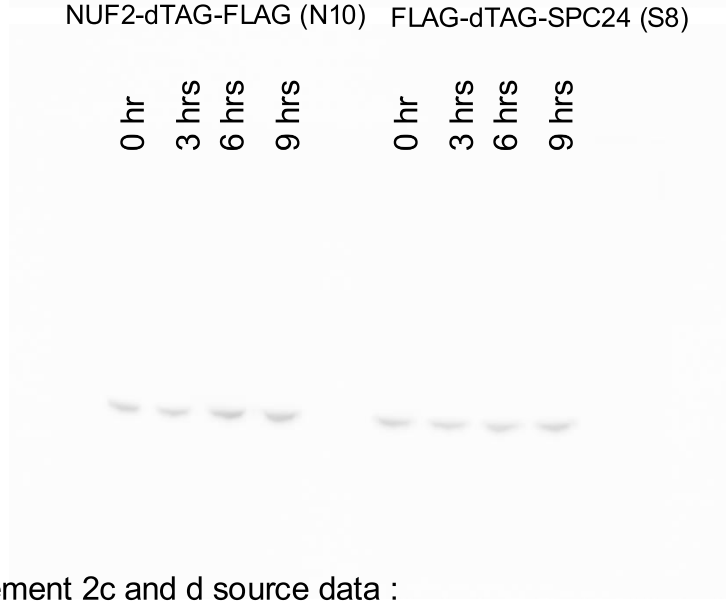

Figure 4-figure supplement 2c and d source data :
Original chemiluminescence images of blot incubated with anti-FLAG (top) and anti β-actin primary antibody (bottom) showing levels of NUF2-dTAG-FLAG or FLAG-dTAG-SPC24 indicated with black and blue arrowheads respectively at indicated time points after addition of dTAG ligands as labelled.

Blot incubated with
anti SPC24
primary antibody

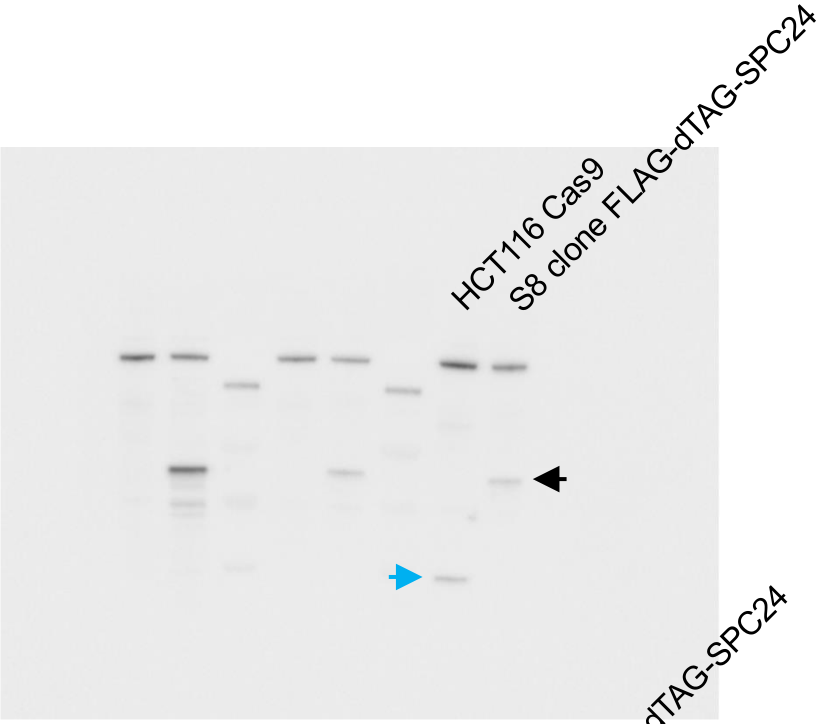

Blot incubated with
anti β-actin
primary antibody

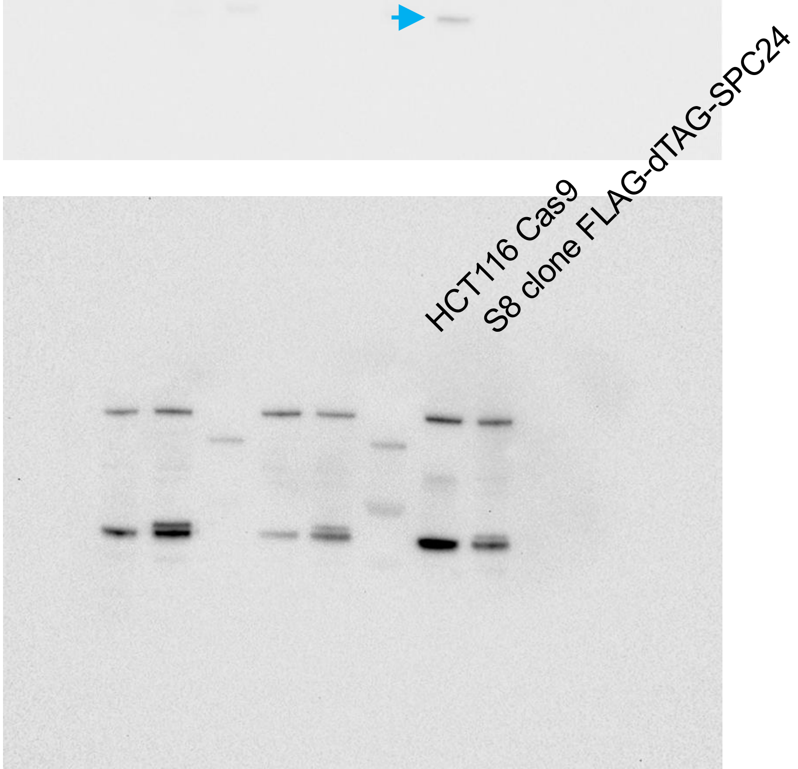

Figure 4-figure supplement 2e source data:
Original chemiluminescence images of blot incubated with anti-SPC24 (top) and anti β-actin primary antibody (bottom) showing levels SPC24 (blue arrowhead) or dTAG-FLAG-SPC24 (black arrowhead) in HCT116 Cas9 and S8 cell lines as indicated. Lanes that are not labelled in this blot are unrelated to S8e.

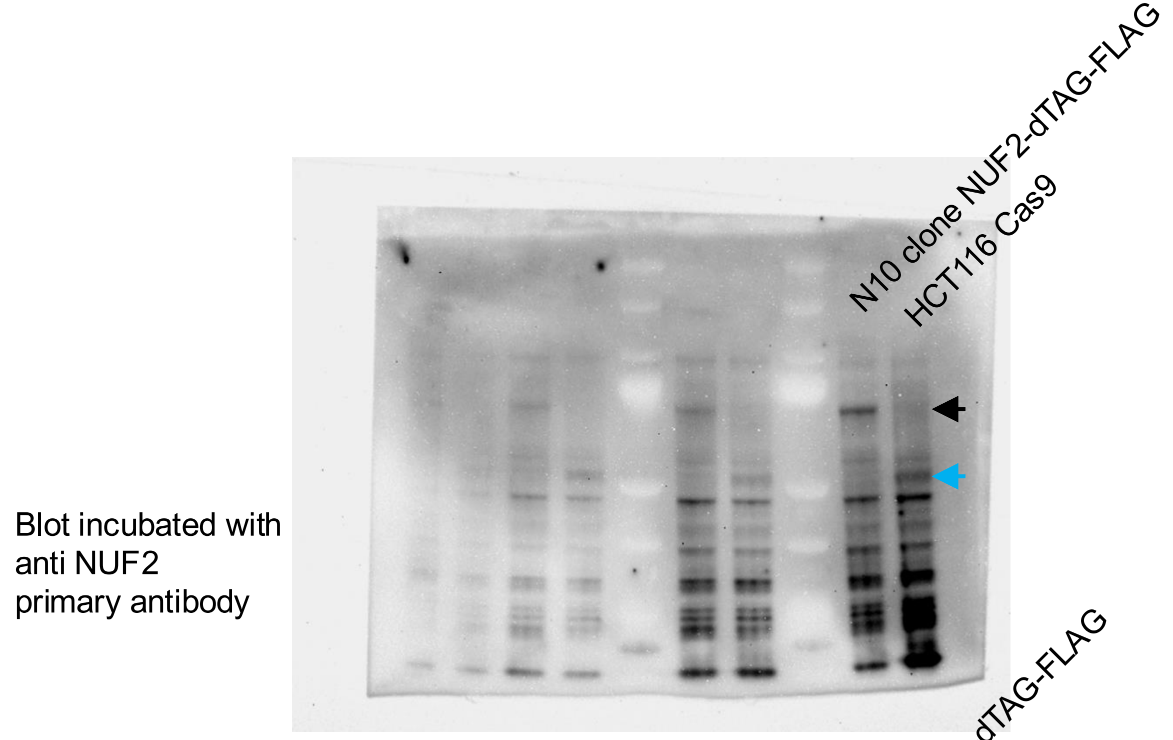

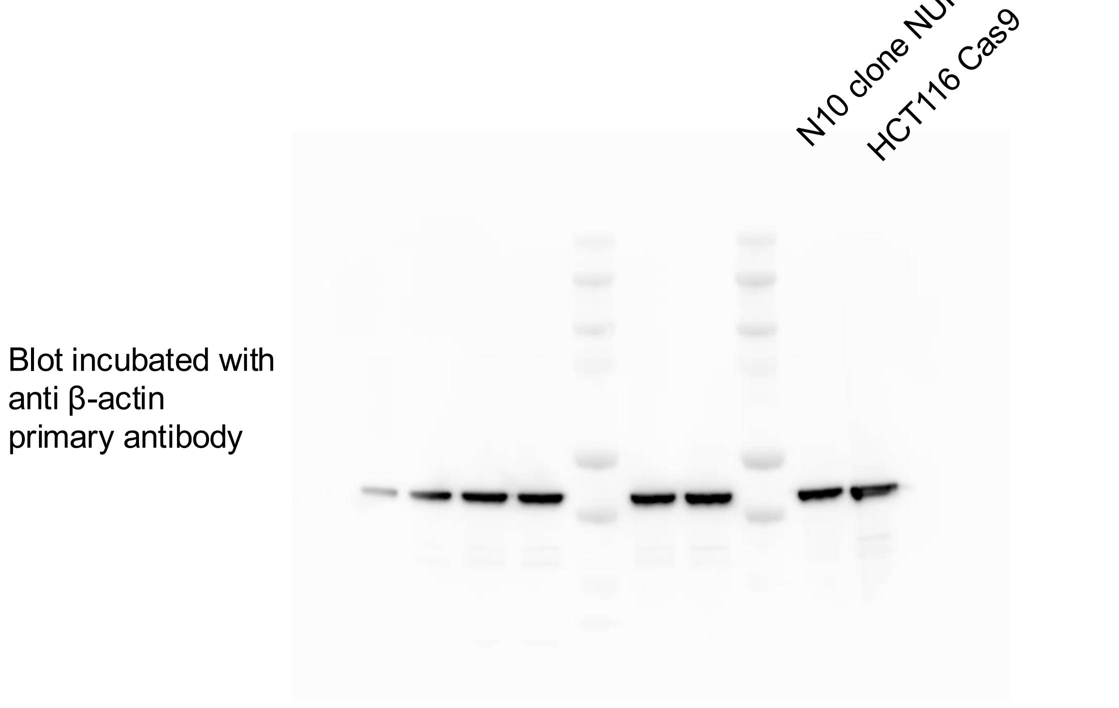

Figure 4-figure supplement 2f source data:
Original chemiluminescence images of blot incubated with anti-NUF2 (top) and anti β-actin primary antibody (bottom) showing level of NUF2 (blue arrowhead) or NUF2-dTAG-FLAG (black arrowhead) in HCT116 Cas9 and N10 cell lines as indicated. Lanes that are not labelled in this blot are unrelated to S8f.
