# Supplementary material for: Orderly mitosis shapes interphase genome architecture
Source: eLife. 2026 Apr 21;14:RP108410. doi: 10.7554/eLife.108410 (PMC13099139; doi:10.7554/eLife.108410)
Supplement: Figure 4—figure supplement 3—source data 2. [file elife-108410-fig4-figsupp3-data2.zip › Figure_4_figure_supplement_3_source_data_2.pdf]

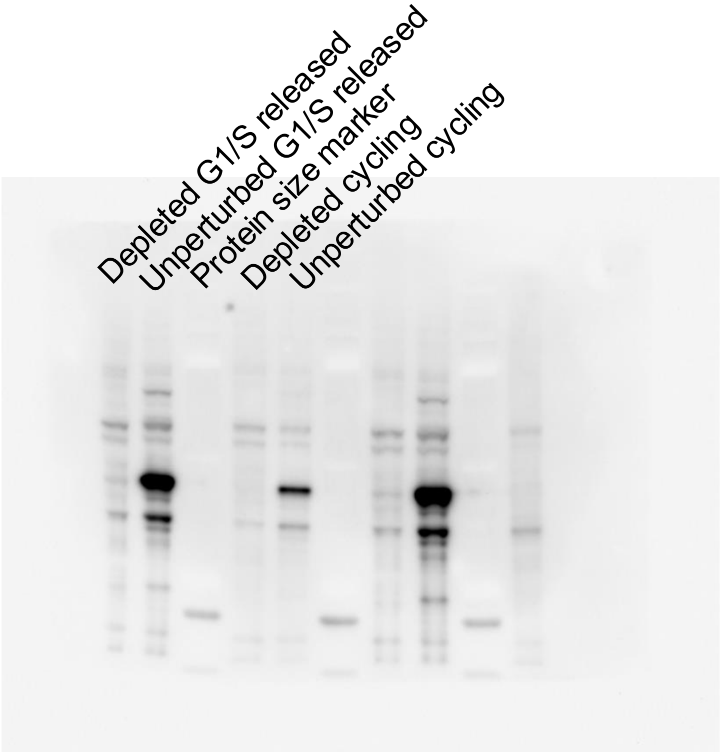

Blot incubated with
anti FLAG
primary antibody

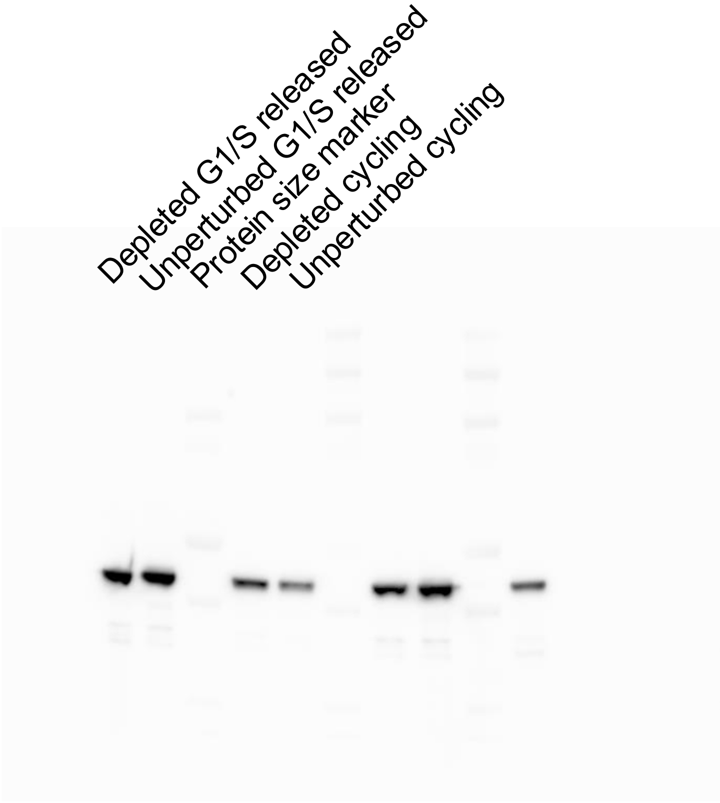

Blot incubated with
anti β-actin
primary antibody

Figure S9a source data:
Original chemiluminescence images of blot incubated with anti-FLAG (top) and anti β-actin primary antibody (bottom) showing levels FLAG-dTAG-SPC24 in G1/S arrested or cycling cells in presence (depleted) or absence (unperturbed) of dTAG ligand as indicated. Lanes that are not labelled in this blot are unrelated to S9a.

Blot incubated with
anti FLAG
primary antibody

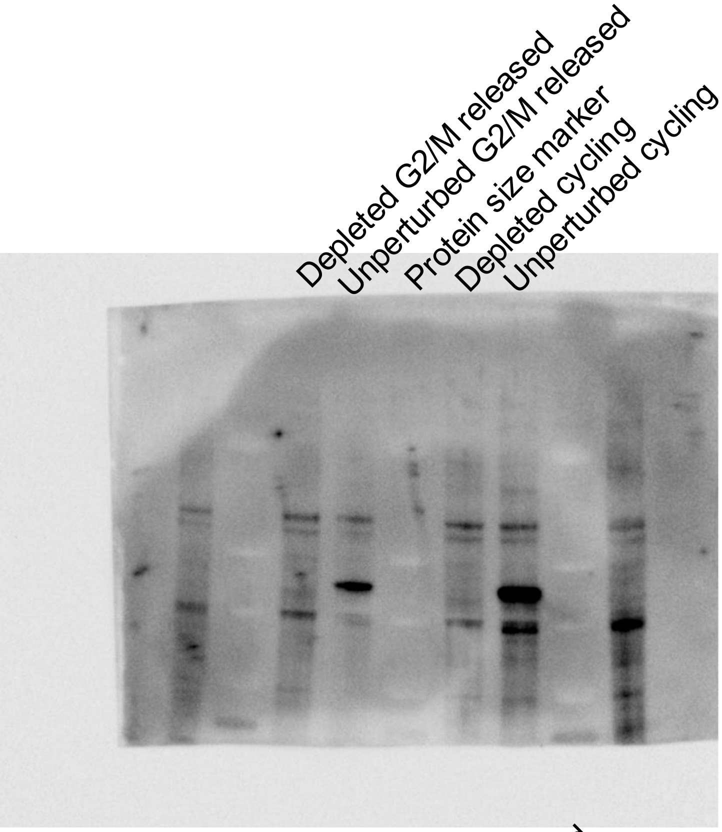

Blot incubated with
anti β-actin
primary antibody

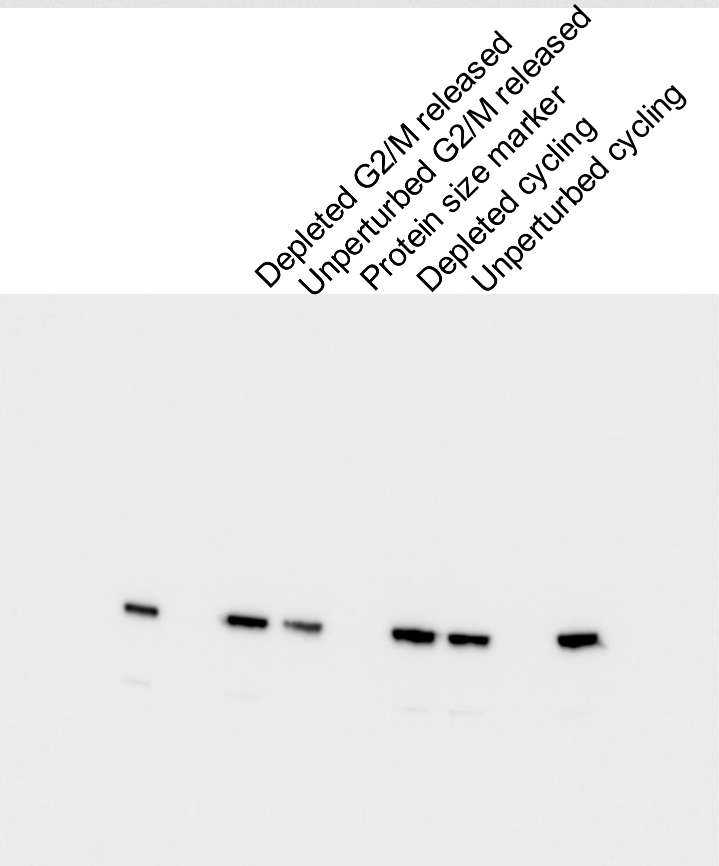

Figure S9b source data:
Original chemiluminescence images of blot incubated with anti-FLAG (top) and anti β-actin primary antibody (bottom) showing levels FLAG-dTAG-SPC24 in G2/M arrested or cycling cells in presence (depleted) or absence (unperturbed) of dTAG ligand as indicated. Lanes that are not labelled in this blot are unrelated to S9a.

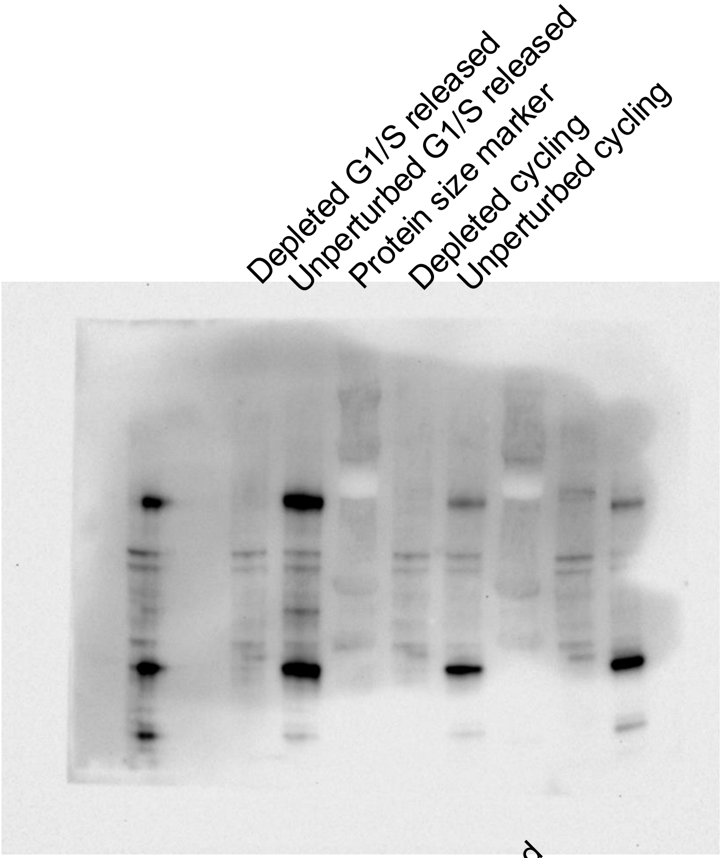

Blot incubated with
anti FLAG
primary antibody

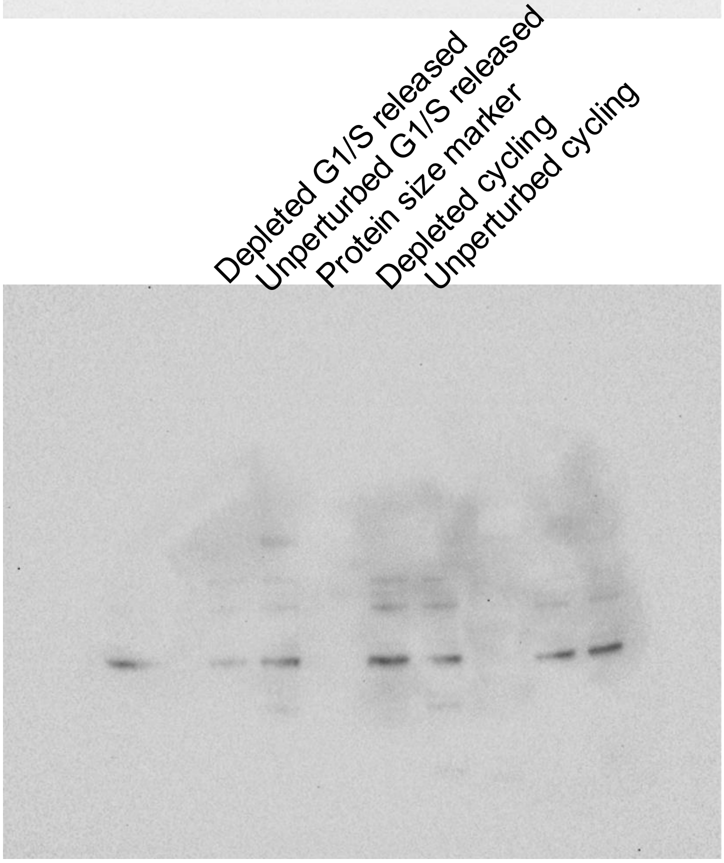

Blot incubated with
anti β-actin
primary antibody

Figure S9c source data:
Original chemiluminescence images of blot incubated with anti-FLAG (top) and anti β-actin primary antibody (bottom) showing levels NUF2-dTAG-FLAG in G1/S arrested or cycling cells in presence (depleted) or absence (unperturbed) of dTAG ligand as indicated. Lanes that are not labelled in this blot are unrelated to S9a.

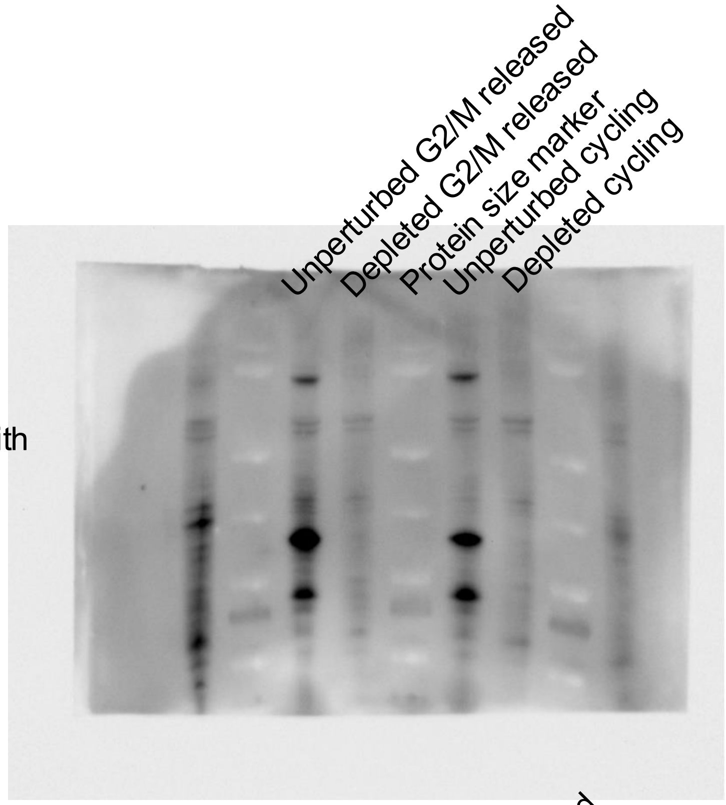

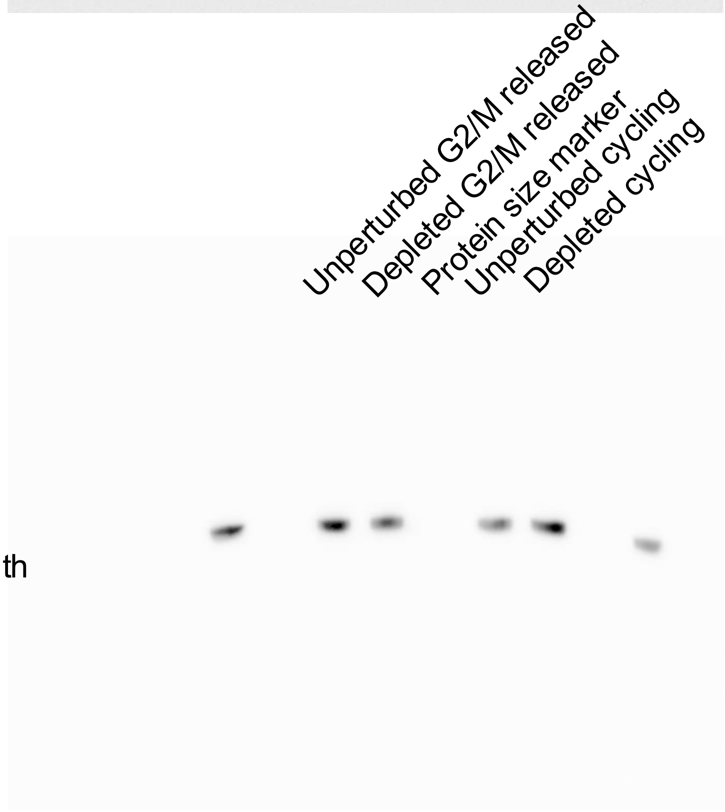

Figure S9d source data:
Original chemiluminescence images of blot incubated with anti-FLAG (top) and anti β-actin primary antibody (bottom) showing levels NUF2-dTAG-FLAG in G2/M arrested or cycling cells in presence (depleted) or absence (unperturbed) of dTAG ligand as indicated. Lanes that are not labelled in this blot are unrelated to S9a.
